# Conservatively treated glassy cell carcinoma of the cervix

**DOI:** 10.1186/1477-7819-6-92

**Published:** 2008-08-28

**Authors:** Gabriella Ferrandina, Vanda Salutari, Marco Petrillo, Arnaldo Carbone, Giovanni Scambia

**Affiliations:** 1 Department of Oncology, Catholic University, Campobasso, Rome, Italy; 2 Gynecologic Oncology Unit, Catholic University, Rome, Italy; 3 Institute of Human Pathology, Catholic University, Campobasso, Rome, Italy

## Abstract

**Background:**

Very little data about the conservative treatment of early stage glassy cell cervical cancer have been reported.

**Case presentation:**

A 30-year old patient, nulligravida was admitted to the Gynecologic Oncology Unit of the Catholic University of Campobasso for irregular post-coital vaginal bleeding. The patients was staged as having FIGO stage IB1 (tumor diameter = 2 cm) squamous cervical cancer. After extensive counseling of the patient and her family, laparoscopic pelvic lymphadenectomy and cold knife conization were performed. The final diagnosis was FIGO Stage IB1 glassy cell carcinoma. Currently, after a follow-up of 38 months, she has no evidence of disease.

**Conclusion:**

We reported a case of early stage glassy cell cancer patient, who was conservatively treated by conization and laparoscopic pelvic lymphadenectomy.

## Background

Over the past decade, the treatment of cervical cancer has evolved registering a gradual abandonment of radical surgery in favor of more conservative approaches: this becomes even more relevant considering that approximately 15% of all cervical cancers, and 45% of surgically treated stage IB cervical cancers occur in women < 40 years of age [1]. These figures are expected to increase due to the widespread use of cervical cancer screening which results in overall younger age and an earlier stage of disease at diagnosis. In addition, more and more frequently women defer childbearing, so that an increasing number of women would be diagnosed cervical cancer before having started or completed their reproductive program. Among the uterus preserving techniques, radical vaginal trachelectomy (RVT) with laparoscopic pelvic lymphadenectomy [2] has gained acceptance over the years by the gynecologic oncology community due to the favorable results in terms of oncological and obstetrical outcome [3].

Among the strict criteria employed in the selection of cases who can potentially be offered uterus preserving approaches, tumor histology *per se *seems not to be a relevant factor [4], with the exception of rare histological types such as adenosquamous, neuroendocrine tumors or glassy cell carcinomas which have been generally associated with a higher risk of recurrence [5,6], and considered a contraindication to conservative treatment [7,8]. In particular, glassy cell carcinomas first described by Glücksmann and Cherry [9] in the uterine cervix, are typically composed of malignant cells showing a moderate amount of cytoplasm with "ground glass" appearance, distinct cell membranes stained with eosin or periodic acid-Schiff, and large nuclei with prominent nucleoli. These tumors have been considered since the beginning as an uncommon variant of poorly differentiated adenosquamous carcinoma [9], endowed with resistance to radiation therapy and unfavorable prognosis [10].

To our knowledge, only three cases of glassy cell carcinomas undergoing conservative treatment by laparoscopic pelvic lymphadenectomy and radical vaginal trachelectomy have been reported [11].

Here, we report the case of a stage IB1 cervical glassy cell carcinoma patient, who was safely treated with cold knife conization plus laparoscopic pelvic lymphadenectomy.

## Case presentation

A 30-year old patient, nulligravida was admitted in March 2005, to the Gynecologic Oncology Unit of the Catholic University of Campobasso, for irregular post-coital vaginal bleeding. Her medical history was unremarkable. Her gynecological history was negative with menarche at the age of 12 years, and regular menses until 6 months before the occurrence of the symptoms.

Gynaecological examination revealed a normal size uterus, and no adnexal masses. A circumscribed, ulcerated lesion (maximum diameter = 2 cm) was documented in the posterior esocervix. Parametria and vagina appeared uninvolved. Colposcopy-guided biopsy and curettage of endocervical canal were performed revealing an invasive squamous cell cervical carcinoma with areas of poor differentiation. Transabdominal and transvaginal ultrasound examination documented the presence of a normal size uterus showing normal echogenicity with the exception of a vascularized hypoechogenic area (18 × 14 × 11 mm) located in the cervix.

Staging evaluation including chest X-ray, total body CT scan, and pelvic magnetic resonance imaging (MRI) documented the presence of a tumor mass (maximum diameter = 2 cm) located in the uterine cervix, and no enlarged lymph nodes. Examination under anesthesia revealed an ulcerated lesion of maximum diameter of 2 cm, without vaginal and parametrial involvement. Squamous cell carcinoma antigen levels were negative. The patient was staged as having FIGO stage IB1 cervical cancer.

After extensive counseling of the patient and her family, she opted for a conservative approach. Open laparoscopy was carried out: peritoneal washing and a careful inspection of the adnexae and intra abdominal organs was performed. Systematic pelvic lymphadenectomy was performed up to internal iliac lymph nodes, and they returned as negative at frozen section examination. Several biopsies of the vaginal walls were obtained; these were negative for disease on frozen section. A cold knife conization was performed, and frozen section analysis showed that the lateral and deep margins of the tissue specimen were uninvolved. The biopsy of the endocervical canal also resulted negative at frozen section.

**Figure 1:**
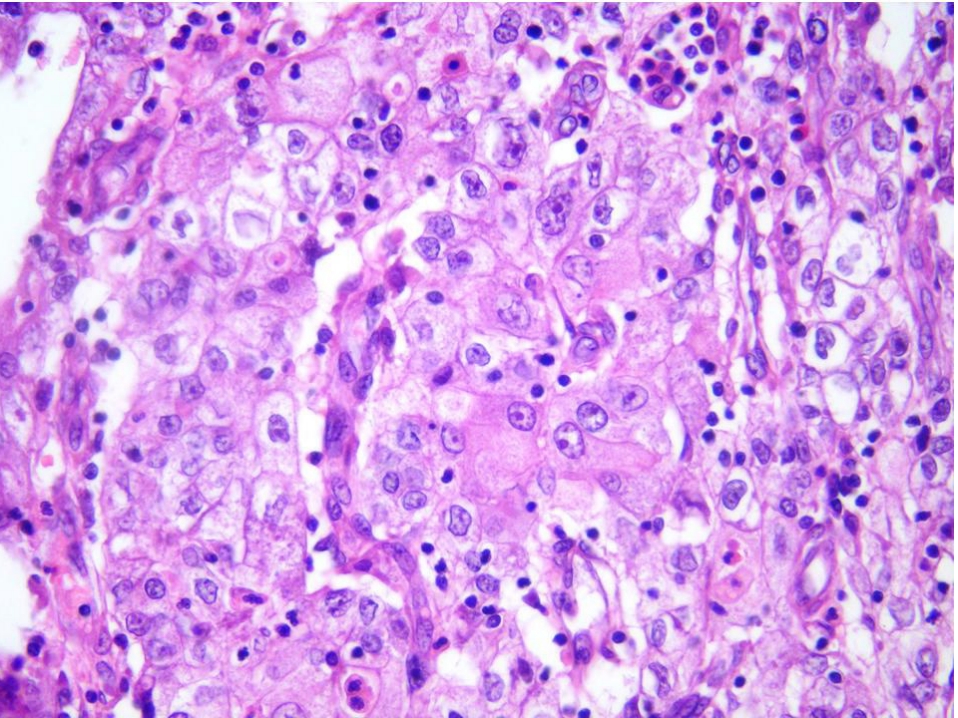
**Glassy cell carcinoma of the cervix: the undifferentiated, glassy cells display large nuclei with prominent nucleoli and granular cytoplasm.** Areas of abundant eosinophils infiltration are present. (Hematoxylin & Eosin, magnification: 200×).

At definitive pathological examination, a nodular lesion of maximum diameter of 2.0 cm (width extension) located in the cone (height = 2 cm, width = 3 cm), was detected. Microscopic examination revealed a tumor composed of nests of large cells with large eosinophilic cytoplasm presenting a ground-glass appearance (Figure 1). Cell membranes were easily recognizable, and tumor nuclei appeared large, presenting prominent nucleoli, and also areas of abundant eosinophil infiltration were present. The tumor showed a stromal invasion of 8 mm out of 1.7 stromal thickness. The lateral and deep margins of the cone were uninvolved for at least 9 mm. All peritoneal biopsies, as well as pelvic lymph nodes (n = 18) were negative. No lymphovascular space involvement was observed. The final diagnosis was FIGO Stage IB1 poorly differentiated carcinoma with > 90% of the tumor represented by neoplastic cells with glassy cell features. A second pathologist, blinded to the first's impression confirmed the diagnosis. Given the rarity of this histological type and its prognostic features, therapeutic options including radical trachelectomy, hysterectomy, or adjuvant treatment were carefully discussed with the patient, who nevertheless decided to undergo only strict follow-up procedures. The patient was then followed with gynecological examination, pap smear, and colposcopy every 3 months for the first 2 years, and every 6 months thereafter, and was also requested to perform chest x-ray and pelvic MRI every year. Currently, after a follow up of 38 months, she has no evidence of disease.

Cervical stenosis was documented after 21 months since surgery, and was easily managed by cannulation of the cervical canal under anesthesia.

## Discussion

We report a case of early stage glassy cell cancer in a patient, who was conservatively treated by conization and laparoscopic pelvic lymphadenectomy. Indeed, among the fertility preservation approaches to early stage cervical carcinoma, RVT has gained much attention because of the recognized oncologic efficacy and safety. Intra- and postoperative complications have been reported to be approximately 4% and 12% of cases, respectively [8], and even less radical procedures such as conization plus laparoscopic pelvic lymphadenectomy have been investigated in selected cases of stage IB1 squamous cell carcinoma < 2 cm diameter [7]. While the fertility preserving procedures are widely accepted for tumors with squamous histological type, and also adenocarcinomas, which *per se *should not be considered a contraindication to conservative treatment, some concerns have been raised for rare histological types such as adenosquamous, neuroendocrine or glassy cell carcinomas. In particular, conservatively treated neuroendocrine and adenosquamous tumors have been reported to carry out a very unfavorable prognosis [5,6]. On the other hand, very few data about early stage glassy cell cervical cancer have been reported: of 3 cases treated with laparoscopic pelvic lymphadenectomy and RVT, all were reported as having no evidence of disease at time of publication [11]. No case of early stage glassy cell carcinoma treated with conization plus laparoscopic pelvic lymphadenectomy has been reported until now.

Despite the extensive counseling about the possibility to perform trachelectomy or adjuvant treatment after final diagnosis, our patient decided only to undergo strict follow-up procedures, and is currently without evidence of disease after 38 months since initial diagnosis.

## Conclusion

We report a case of an early stage glassy cell cervical carcinoma patient, who was successfully treated with conization and laparoscopic pelvic lymphadenectomy. Given the rarity of this tumor histological type, and the paucity of data about its natural history, which has been reported to be similar to other histological types only with the employment of multimodal treatment strategies [12], caution should be taken to i) carefully evaluate the patients' fertility potential; ii) extensively counsel the patients about the risk/benefit of a conservative treatment; iii) investigate the patients' compliance to undergo strict follow-up procedures.

## Competing interests

The authors declare that they have no competing interests.

## Authors' contributions

GF conceived of the study, participated in its design and drafting. VS participated in the design of the study and collected the clinical data. MP participated in the design of the study and collected the clinical data. AC carried out the histopathological evaluation. GS conceived of the study, and participated in its design and coordination and helped to draft the manuscript. All authors read and approved the final manuscript.

## Consent

Written informed consent was obtained from the patient for publication of this case report and any accompanying images. A copy of the written consent is available for review by the Editor-in-Chief of this journal.
